# The barriers and facilitators of developing clinical competence among master’s graduates of gerontological nursing: a qualitative descriptive study

**DOI:** 10.1186/s12909-022-03553-x

**Published:** 2022-06-27

**Authors:** Negin Masoudi Alavi, Zohreh Nabizadeh‑Gharghozar, Neda Mirbagher Ajorpaz

**Affiliations:** 1 Trauma Nursing Research Center, Kashan University of Medical Sciences, Kashan, Iran; 2 Student Research Committee, Nursing & Midwifery School, Shahid Behshti University of Medical Sciences, Tehran, Iran; 3 Autoimmune Diseases Research Center, Kashan University of Medical Sciences, Kashan, Iran

**Keywords:** Clinical competence, Conventional content analysis, Master’s course of gerontological nursing

## Abstract

**Background:**

Clinical competence development is a main goal of specialized nursing courses. Nonetheless, some master’s graduates of gerontological nursing programs have inadequate Clinical competence. The aim of this study was to explore the barriers and the facilitators to clinical competence development among the master’s graduates of gerontological nursing.

**Method:**

This qualitative descriptive study was conducted in 2020. Participants were twenty nursing master’s students, master’s graduates, and instructors of gerontological nursing. They were purposively selected from several faculties of nursing and midwifery in Tehran, Isfahan, and Kashan, Iran. Semi-structured interviews were held for data collection and the conventional content analysis proposed by Graneheim and Lundman was used for data analysis. Data were managed using the MAXQDA 10 software.

**Results:**

The main barriers to clinical competence development were students’ neglectfulness towards learning, inefficiency of educational system, and ineffective management. The main facilitators to clinical competence development were effective educational planning and management improvement.

**Conclusion:**

There are different personal, educational, and managerial barriers and facilitators to clinical competence development among master’s graduates of gerontological nursing. Effective educational planning and management improvement are needed for clinical competence development among master’s students and graduates of gerontological nursing.

## Background

Nurses’ clinical competence is a significant factor affecting nursing care quality and patient outcomes. Clinical competence is called knowing in action and is an automated response based on the internalized knowledge [1]. Clinical competence is considered as the final outcome of nursing education and is defined as knowledge, skill, attitude, and ability for safe and effective practice without others’ supervision [2].

According to the texts, the areas of clinical competence in nursing are different. In a study, clinical competence of nursing are divided into five areas: ethical-professional practice, holistic practice, personal communication, care organization, and individual-professional development [3]. Another study introduced the clinical competence of nursing including four areas of professional responsibility, care management, interpersonal relationships and interprofessional care, and quality improvement [4]. The results of a qualitative study also showed that having knowledge, skills and behaviors based on professional performance and moral values ​​are among the clinical competence that geriatric nursing needs to strengthen these competencies to work in the clinic [5].

Clinical competence has direct relationship with patient safety, patient satisfaction, and care quality [6, 7]. Studies showed that care delivery by competent nurses was associated with lower re-hospitalization rate, better management of geriatric syndromes among elderly people [8, 9], closer adherence to dietary regimens, and greater patient and family satisfaction [10]. On the other hand, a large-scale retrospective study in Europe revealed that care delivery by nurses with low clinical competence was associated with higher mortality rate [11]. Clinical competence has positive outcomes for nurses too. Studies reported that clinical competence had inverse relationship with job burnout [12] and positive relationship with quality of working life [13], self-efficacy, professional confidence, and effective use of clinical skills [14]. Clinical competence development for optimum nursing practice starts during nursing education [15] and continues during actual practice, effective communications, and knowledge transfer to practice at workplace [16]. Consequently, nursing education should focus on developing nursing students’ clinical competence for independent practice in complex clinical environments [17].

Despite the importance of clinical competence to quality care delivery and patient outcomes, previous studies showed that some nurses and nursing graduates had inadequate clinical competence. For example, 76.6% of nursing faculty members in a study in Iran reported that master’s graduates had limited clinical competence for clinical nursing practice and 92.97% of them highlighted the necessity of specialized clinical courses for master’s students [18]. Nursing master’s graduates in a study in Italy also believed that nursing authorities mainly focused on educational, managerial, and research-related skills and paid limited attention to the development of students’ clinical skills [19].

There are many different challenges to clinical competence development among master’s nursing students and graduates [20]. A study reported leadership style, educational system, quality of working life, organizational learning, and organizational support as managerial and organizational factors affecting nurses’ clinical competence development [21]. Several other studies also reported that the barriers to clinical competence development among master’s students of gerontological nursing were elderly people’s limited collaboration with and trust in healthcare providers, poor teamwork, interpersonal conflicts, inattention to the humanistic aspects of care, disinterest of experienced instructors in clinical education, limited empathy between instructors and students, high number of students, rapid changes in treatment and care methods, and poor planning for using professional skills such as problem solving, critical thinking, and clinical reasoning [22–25].

Most previous studies explored factors affecting clinical competence development among bachelor’s nursing students and hence, there is limited information about these factors among master’s nursing students. Also in Iran due to the infancy of the field and the first time that the curriculum of the elderly nurse has been developed in the master's degree. Therefore, the present study was conducted to narrow this gap. The aim of the study was to explore the challenges of clinical competence development among master’s graduates of gerontological nursing.

## Method

### Design

This qualitative descriptive study [26] and based on the naturalistic research approach [27] was conducted in 2020.Qualitative research is a way to gain insight through discovering meanings. This insight is not gained by finding the cause-and-effect relationship, but by our perception of the whole. In a holistic framework, qualitative research is a tool for deep, rich and inherently complex exploration of phenomena that insights from this process can guide nursing practice and contribute to the important theory design process in the production of nursing knowledge [28]. This study qualitative descriptive was chosen since this method allows the deep exploration of experience, as well as interpretation of the data, leading to conclusions about the meaning of these experiences.

### Participants and setting

The setting of the study was the Faculties of Nursing and Midwifery of Tehran, Shahid Beheshti, Isfahan, and Kashan Universities of Medical Sciences in Tehran, Isfahan, and Kashan, Iran. Sampling was purposefully done with maximum variation respecting participants’ age, gender, and work experience. Study participants were seven master’s students of gerontological nursing, seven master’s graduates of gerontological nursing, and six gerontological nursing instructors. Inclusion criterion for master’s graduates and nursing instructors was a clinical work experience of at least two years. Voluntary withdrawal from the study was the only exclusion criterion.

### Data collection

Data were collected using semi-structured interviews with questions about the challenges of clinical competence development. Examples of these questions for instructors, students and graduates were (Table 1). Besides main interview questions, probing questions such as “May you explain more about this?” and “Can you provide an example?” were also used to enrich the data. The duration of the interviews was fifty minutes, on average. All interviews were audio-recorded. Study data were saturated with twenty interviews. Theoretical saturation ensues when new data analysis does not provide additional material to existing theoretical categories, and the categories are sufficiently explained [29]. This study was saturated when no new data was distilled from interviews, and the categories could sufficiently cover salient variations and process, and the interrelationships between categories had been delineated appropriately.

**Table 1:** Interview questions

Questions for instructors	Questions for students and graduates
1. May you please explain about your experiences of clinical education for master’s students of gerontological nursing? 2. Can you explain about one of your educational sessions in classroom and in clinical settings? 3. Which challenges do you experience during your teaching for master’s students of gerontological nursing? 4. What measures do you take to develop students’ clinical competence?	1. Is the curriculum of gerontological nursing consistent with expectations from its graduates? 2. What are the strengths and the limitations of the curriculum? 3. What measures can improve the quality of the curriculum? 4. May you please explain about your experience of an educational session which was efficient in your opinion? 5. May you please explain about an educational session which was inefficient in your opinion? 6. What are the barriers to clinical competence development? 7. What are the facilitators to clinical competence development?

### Data analysis

Study data were analyzed concurrently with data collection through conventional content analysis as proposed by Graneheim and Lundman [30]. Initially, interviews were transcribed and perused for several times to immerse in the data. Then, meaning units were identified and coded and the codes were grouped into subcategories and categories according to their similarities [30]. Data were managed via the MAXQDA 10 software.

### Trustworthiness

Guba and Lincoln’s (1986) criteria were used to ensure the accuracy and stability of the research data. The credibility of the data was assessed using member-checking and prolonged engagement techniques. For member checking technique, the participants reviewed the content of the interview and the resulting codes to ensure the accurate meaning and for really reflecting their experiences. The data were also assessed by an external researcher (peer debriefing). To ensure the dependability, data collection methods, interview, taking notes, coding, and data analysis were expressed in detail in order to make judging by the external auditor (external auditing). In order to achieve confirmability, the audit trail method was used, so that all stages of the research, especially the stages of data analysis and the results, were provided to checking of two expert colleagues in the field of qualitative research. The transferability of the findings was also established by providing a rich description of the research report and the content of the interviews was represented by the selected quotations from the participants [31].

## Results

Twenty master’s students of gerontological nursing (*n* = 7), master’s graduates of gerontological nursing (*n* = 7), and gerontological nursing instructors (*n* = 6) participated in this study (Table 2). The mean of their age was 40 ± 8.2 years.

**Table 2:** Participants’ characteristics

No	Gender	Age (years)	Educational level	Work experience (Years)
1	Male	28	Master’s student	3
2	Female	40	Master’s graduate	14
3	Female	50	Instructor	26
4	Female	34	Instructor	6
5	Male	43	Master’s graduate	18
6	Female	33	Master’s student	5
7	Female	40	Instructor	14
8	Female	54	Instructor	23
9	Female	35	Master’s graduate	12
10	Male	60	Master’s graduate	33
11	Male	58	Instructor	18
12	Female	42	Instructor	14
13	Male	56	Master’s graduate	24
14	Female	39	Master’s student	12
15	Female	41	Master’s graduate	13
16	Female	31	Master’s student	6
17	Male	37	Master’s student	15
18	Female	42	Master’s student	6
19	Male	38	Master’s graduate	13
20	Female	29	Master’s student	5

During data analysis, 670 primary codes were generated which were categorized into sixteen subcategories, five main categories, and the two main themes of the barriers and the facilitators to clinical competence development among master’s graduates of gerontological nursing.

### Barriers to clinical competence development

The barriers to clinical competence development among master’s graduates of gerontological nursing came into three main categories, namely students’ neglectfulness towards learning, inefficiency of educational system, and ineffective management (Table 3).

**Table 3:** The subcategories, main categories, and main themes of the study

Subcategories	Categories	Themes
1.1.1. Lack of motivation	1.1. Students’ neglectfulness towards learning	1. Barriers to clinical competence development
1.1.2. Limited knowledge development		
1.1.3. Credentialism		
1.1.4. Routine-based practice		
1.2.1. Incompetence of clinical nursing instructors	1.2. Inefficiency of educational system	
1.2.2. Defective student evaluation		
1.2.3. Shortage of clinical education environment		
1.2.4. Poor educational materials		
1.2.5. Theory–practice-research gap		
1.3.1. Incompetence of managers	1.3. Ineffective management	
1.3.2. Limited job satisfaction		
2.1.1. Competence-based staff recruitment	2.1. Effective educational planning	2. Facilitators to clinical competence development
2.1.2. Developing a need-based educational program		
2.2.1. Effective management	2.2. Management improvement	
2.2.2. Improvement of continuing education		
2.2.3. Improvement of the quality of working life		

#### Students’ neglectfulness towards learning

Students have significant roles in promoting their own learning. However, their neglectfulness towards their learning can reduce the effectiveness of all other teaching–learning activities. The subcategories of this main category were lack of motivation, limited knowledge development, credentialism, and routine-based practice.

##### Lack of motivation

Motivation is a basic drive for learning, without which the best educational programs may be fruitless. Participants’ experiences showed that factors such as nursing instructors’ negative attitudes towards nursing, ambiguities about the future of gerontological nursing, instructors’ limited professional competence, nursing staff’s mistreatment of nursing students and instructors, and poor public image of gerontological nursing can contribute to nursing students’ lack of motivation.

Some recruitment advertisements announce that they need nurses for looking after elderly people. However, they recruit persons who may not have even a high school diploma. Such practice definitely devalues nursing and reduces our motivation (P. 8).

##### Limited knowledge development

The major reasons of running graduate courses in nursing are ever-changing needs of communities and the profession, care quality issues, and rapid advances in technology. However, participants reported poor knowledge development among nursing students due to factors such as limited time for studying main textbooks due to clinical and thesis-related activities, sufficing to pamphlets, passive participation in classrooms, and studying just for exams. These factors result in superficial learning.

I’m a master’s student and simultaneously do my mandatory post-graduation service. My work shifts are so many that I have no time for completely studying a gerontological nursing textbook. Therefore, I refer to textbooks just to do my assignments. Certainly, I don’t have adequate academic competence for nursing practice (P. 16).

##### Credentialism

One of the main goals of graduate courses of nursing is to develop students’ clinical skills. However, the experiences of some participants’ showed that they had opted for graduate education in order to distance from clinical nursing practice, obtain better career advancement opportunities, and have higher income.

I think students do not like to develop their knowledge and promote their learning. They find classrooms boring and like their master’s course to finish sooner and obtain their degree as soon as possible (P. 11).

##### Routine-based practice

Routine-based practice has been a serious problem in nursing since many years ago. Novice nurses need to adhere to ward routines so closely that they may gradually put aside their professional knowledge and imitate colleagues’ behaviors, obey their orders, get neglectful towards patients’ needs, and resort to routine-based care instead of patient-centered care.

Currently, we have some staff in our wards who have routine-based practice and never care whether their services are based on patients’ needs (P. 12).

#### Inefficiency of educational system

The educational system of nursing should provide nurses with adequate knowledge, professional skills, and professional competence for quality care delivery. Nonetheless, most participants noted that the master’s course of gerontological nursing had serious shortcomings and reported its inefficiency as a major barrier to clinical competence development. This main category had five subcategories, namely incompetence of clinical nursing instructors, defective student evaluation, shortage of clinical education environment, poor educational materials, and theory–practice-research gap.

##### Incompetence of clinical nursing instructors

Clinical instructors have significant role in developing students’ clinical competence. Participants noted that instructors’ limited professional knowledge, limited clinical experience, limited attention to clinical education, and limited responsibility towards student learning negatively affect the process of clinical competence development.

Instructors have neither adequate knowledge nor adequate experience about gerontological nursing. Therefore, they can’t understand elderly people’s needs and can’t provide us with appropriate education (P. 5).

##### Defective student evaluation

Educational evaluation provides data about the outcomes of teaching–learning process to ensure learners’ competence. Participants’ experiences showed inconsistent evaluation criteria and inattention to practical skills of students during evaluation which resulted in superficial learning.

Competition in our class is so intense that the difference among our grade point averages is very small. My classmates have studied a lot for exams and obtained good scores; however, if you ask them questions about the courses of the last term, they can’t remember anything or they don’t know how to manage an elderly person with stroke (P. 2).

##### Shortage of clinical education environment

Nursing is a practical profession in which students obtain practical skills and develop their professional competence in clinical environment. However, participants referred to the shortage of appropriate clinical education environments as a main barrier to clinical competence development.

Normally, learners should attend clinical environments to promote their learning through direct observation and role modeling. However, there is a shortage of clinical settings for education in our country (P. 3).

##### Poor educational materials

Graduate courses in nursing should empower students for advanced nursing care, develop their professional knowledge, and develop their abilities to play significant roles in nursing practice and nursing education. Nonetheless, participants noted that the master’s course of gerontological nursing does not empower students for advanced care delivery due to poor educational materials, repetition of undergraduate courses, and shortage of quality gerontological nursing textbooks and resources.

Although they expect us to be more professional in clinical practice, our graduate course prepares us for research not for clinical practice (P. 6).

##### Theory–practice-research gap

Nurses need to use research-based data in professional decision making, clinical practice, and interaction with their clients. However, our participants’ experiences showed the limited applicability of educational materials, poor relationship between nursing research and practice, limited use of thesis findings, and the time-consuming process of doing master’s thesis.

These researches we do have no real application. We just waste our time in this two-year master’s course (P. 2).

#### Ineffective management

Ineffective management can cause serious damages to all parts of an organization. The two subcategories of this main category were incompetence of managers and limited job satisfaction.

##### Incompetence of managers

Meritocracy refers to the selection of managers based on their competence and abilities. Participants’ experiences revealed that the selection of incompetent managers can reduce the quality of organizational activities and result in organizational injustice, relationship-based employments, negligence towards staff’s competencies, reduced job motivation, and staff’s limited use of their abilities.

Unfortunately, managers are selected based on relationships rather than their managerial knowledge and experience. This is a relationship-based selection rather than meritocracy (P. 16).

##### Limited job satisfaction

Participants noted that employment as a staff with bachelor’s degree despite having master’s degree, dissatisfaction with the status of gerontological nursing, heavy workload, unfair payments, managers’ limited attention to care quality, and their greater attention to non-professional affairs can reduce productivity, motivation, and care quality, and thereby, restrict clinical competence development.

Elderly care centers should be managed by gerontological nurses because they have the necessary knowledge and skills. Nonetheless, gerontological nurses’ position is still unknown (P. 3).

### Facilitators to clinical competence development

The second main theme of the study was the facilitators to clinical competence development. The two main categories of this main theme were effective educational planning and management improvement (Table 3).

#### Effective educational planning

According to the participants, the efficiency of educational system largely depends on effective educational planning and the curriculum of nursing should be developed based on changes in clinical settings and evidence-based data. This main category had two subcategories, namely competence-based staff recruitment and development of a need-based educational program.

##### Competence-based staff recruitment

Recruitment of competent staff is a key factor in organizational success. Participants noted that besides adequate professional knowledge, applicants for gerontological nursing master’s programs need to have adequate clinical work experience, great professional interest, appropriate personality characteristics, and ability to work with elderly people.

If we are going to have competent nurses, we need to use better recruitment policies (P. 11).

##### Developing a need-based educational program

Need assessment provides valuable data for goal setting and an appropriate context for managing financial and human resources. Therefore, participants highlighted that the curriculum of gerontological nursing should be revised based on the needs of students and the expectations of elderly people.

We need to perform need assessment to identify the needs and the characteristics of elderly people (P. 10).

#### Management improvement

Participants’ experiences showed that managers’ leadership style can affect teaching–learning environment and clinical competence development. This main category had three subcategories, namely effective management, improvement of continuing education, and improvement of nurses’ quality of working life.

##### Effective management

According to the participants, managers need to have the ability to guide, supervise, coordinate, and facilitate organizational affairs, support changes, develop clear job specifications, and improve communications and workplace environment.

Managers’ feedback to care services has significant role in improving my practice. Without control, how can I know about my mistakes (P. 7)?

##### Improvement of continuing education

Continuing education is a continuous process of professional development which aims at improving staff’s knowledge and skills. Participants noted that continuing education programs can improve care quality, shorten patients’ hospital stay, and update staff’s professional knowledge and skills.

Continuing education is necessary for competence development. With greater knowledge and skills, I can provide better education to my patients. Patient education in turn shortens patients’ hospital stay and reduces re-hospitalization rate (P. 5).

##### Improvement of the quality of working life

According to the participants, factors such as adequate salaries, appropriate encouragements and rewards, appropriate safety and welfare facilities, and managerial support can improve nurses’ quality of working life, reduce their occupational stress, improve their job satisfaction, and encourage them for CC development.

Nursing care delivery to elderly people is associated with high levels of occupational stress and hence, it needs adequate psychological support. For example, managers need to provide nurses with recreational and educational facilities in order to improve their job motivation (P. 9).

## Discussion

This study aimed at assessing the challenges of clinical competence development among master’s graduates of gerontological nursing. Findings revealed two main themes, namely the barriers and the facilitators to clinical competence development. The barriers to clinical competence development were students’ neglectfulness towards learning, inefficiency of educational system, and ineffective management. In line with these findings, a previous qualitative study reported that the causes of limited clinical competence from the perspectives of clinical instructors and nursing students were lack of human and material resources (with the subcategories of staff shortage, consideration of students as staff, and lack of clinical equipment), staff burnout (with the subcategories of low morale, negative attitude, and lack of recognition, support, and incentives), and lack of quality control (with the subcategories of lack of continuing education, lack of feedback, lack of appropriate qualifications, and lack of adequate staffing) [32].

Students’ lack of motivation, as a subcategory of the students’ neglectfulness towards learning main category, was a major barrier to clinical competence development. According to the Vroom’s Expectancy Motivation Theory, the level of students’ motivation for a specific job can affect their career choice behaviors and decisions [33]. Job motivation is a significant factor in professional development and job satisfaction [34]. Students in some previous studies described elderly care as boring, depressing, and arduous and did not consider it as a good career choice [35, 36]. Moreover, the poor quality of clinical environment makes people not consider gerontological nursing as a profession and thereby, reduces students’ motivation for gerontological nursing [37].

Limited knowledge development was another subcategory of the students’ neglectfulness towards learning main category. Gerontological nursing is essential for care delivery to elderly people; however, most nurses may have inadequate knowledge for care delivery to elderly people [38]. Theoretical knowledge is a catalyst for clinical competence development [39]. Therefore, inattention to knowledge development is definitely a major barrier to clinical competence development. Education during work was a reason for limited knowledge development in the present study. A former qualitative study also showed part-time paid employment during education as a factor with negative effects on students’ academic performance [40]. Two other studies reported that students had negative attitudes towards research and considered it boring, difficult, and time-consuming [41, 42].

Credentialism was the third subcategory of the students’ neglectfulness towards learning main category. A study reported that students’ motives for choosing gerontological nursing as a field of study were their motivation to have a higher degree, their motivation to have better access to more career advancement opportunities, and the greater likelihood of successfully passing the entrance exam of the gerontological nursing master’s course. These motives result in superficial learning and limited competence for elderly care [43]. Routine-based practice was the other subcategory of the students’ neglectfulness towards learning main category in the present study. Currently, routine-based practice is a serious challenge in nursing and many efforts are made to substitute it with patient-centered holistic care [44].

The second main category of the barriers to clinical competence development was the inefficiency of educational system. Incompetence of clinical nursing instructors was one of the subcategories of this main category. Clinical instructors’ incompetence has many different reasons such as limited professional knowledge, limited professional interest, limited job motivation, limited perceived support, limited educational and clinical work experience, inattention to clinical education, and limited professional responsibility [45, 46].

Defective student evaluation was the second subcategory of the inefficiency of educational system main category. In line with this finding, previous studies reported inconsistency in student evaluation procedures as well as students’ dissatisfaction with their instructors’ student evaluation [47, 48]. A study also reported greater significance of summative evaluation, inattention to students’ practical skills, inconsistent evaluation criteria, and clinical instructors’ poor performance as the subcategories of inefficient clinical evaluation [47]. Evaluation plans should focus on performance and should be clear, fair, and consistent with standards and learning objectives [49].

The third subcategory of the inefficiency of educational system main category was shortage of clinical education environment. Previous studies also showed lack of specialized learning and caring environments as a major barrier to clinical competence development among maser’s students of gerontological nursing [22, 50]. Most nursing students have problems in clinical practice due to their limited clinical experience, limited opportunities for knowledge transfer to practice, and unfamiliarity with complex clinical environments [51, 52]. These problems and their contributing factors are associated with poor clinical competence and increase students’ anxiety [51]. However, a study reported that students who pass their clinical courses in gerontological care centers are more likely to choose gerontological nursing as a field of study [53].

Study findings also revealed theory–practice gap as another subcategory of the inefficiency of educational system main category. This is in line with the findings of previous studies [54, 55]. A study also showed that the barriers to nurses’ use of research findings were their limited trust in research findings, ambiguities in the conclusions of researches, contradictory results of different researches, and unfamiliarity with English language [56].

The third main category of the barriers to clinical competence development was ineffective management with the two subcategories of managers’ incompetence and limited job satisfaction. In line with this finding, a previous study reported that meritocracy, managers’ leadership style, and managers’ actual practice can affect gerontological nurses [21]. Moreover, several other studies on nurses reported significant direct relationships between clinical competence and job satisfaction [57, 58], between job dissatisfaction and low income, heavy workload, and night shift work [59], and between satisfaction with income and job motivation [60].

We also found effective educational planning and management improvement as the main categories of the facilitators to clinical competence development. Competence-based staff recruitment was one of the subcategories of the effective educational planning main category. A study reported that the personal characteristics of staff can affect the quality of elderly care and the satisfaction of elderly people with care [61]. Moreover, we found that need-based education was another subcategory of the effective educational planning main category. In line with this finding, a former study showed that need-based education with emphasis on learners’ weaknesses promotes learning [62].

Study findings also showed effective management as a subcategory of the management improvement main category. Hospital managers need to be committed to elderly-friendly care and develop policies, procedures, physical environment, and workforce resources based on elderly people’s needs in order to facilitate nursing care delivery to them [63]. However, unclear roles and job specifications can result in interpersonal conflicts among healthcare providers and reduce the effectiveness of care services [64]. We also found improvement of continuing education as another managerial facilitator to clinical competence development. This is in line with the findings of a former study which showed that a continuing education program for novice nurses facilitated their professional development and significantly improved their clinical reasoning skills, care quality, and patient safety [65]. Improvement of the quality of working life was the third subcategory of the management improvement main category of the facilitators to clinical competence development. A study in Korea also showed a significant positive relationship between nurses’ clinical competence and their quality of working life [12].

The main limitation of this study was our limited accessibility to eligible master’s graduates of gerontological nursing and top gerontological nursing authorities for inclusion in the study.

## Conclusion

This study reveals that there are different personal, educational, and managerial barriers and facilitators to clinical competence development among master’s graduates of gerontological nursing. The findings of this study can be used to develop strategies for facilitating clinical competence development among master’s students and graduates of gerontological nursing. These strategies include recruitment interview to determine and select the most appropriate students for gerontological nursing, creation of an appropriate educational setting, development of specialized elderly care centers, reduction of staff shortage and workload by recruiting competent nurses, reduction of nurses’ work hours, timely payment for nurses, interdisciplinary education and continuing education programs, development of clear job specifications for nurses, use of supportive leadership style, and provision of nurses with stronger organizational support.

## Data Availability

The interview dataset generated and analysed during the current study are not publicly available due to promises of participant anonymity and confidentiality. However, on reasonable request the data could be available from the corresponding author. All applications should be sent to nabizadehfaezeh85@yahoo.com. All requests will be answered within a maximum of 1 month by email.
